# Lack of Brain Serotonin Affects Feeding and Differentiation of Newborn Cells in the Adult Hypothalamus

**DOI:** 10.3389/fcell.2019.00065

**Published:** 2019-04-26

**Authors:** Marike van Lingen, Maria Sidorova, Natalia Alenina, Friederike Klempin

**Affiliations:** ^1^Department of Anatomy and Neurosciences, VU Medical Centre, Vrije Universiteit Amsterdam, Amsterdam, Netherlands; ^2^Berlin Institute of Health, Charité – Universitätsmedizin Berlin, Berlin, Germany; ^3^The School of Life Sciences, Immanuel Kant Baltic Federal University, Kaliningrad, Russia; ^4^Max Delbrück Center for Molecular Medicine, Berlin, Germany

**Keywords:** hypothalamus, BrdU, 5-HT, Tph2, NG2, Western-type diet

## Abstract

Serotonin (5-HT) is a crucial signal in the neurogenic niche microenvironment. Dysregulation of the 5-HT system leads to mood disorders but also to changes in appetite and metabolic rate. Tryptophan hydroxylase 2-deficient (*Tph2^-/-^*) mice depleted of brain 5-HT display alterations in these parameters, e.g., increased food consumption, modest impairment of sleep and respiration accompanied by a less anxious phenotype. The newly discovered neural stem cell niche of the adult hypothalamus has potential implications of mediating stress responses and homeostatic functions. Using *Tph2^-/-^* mice, we explore stem cell behavior and cell genesis in the adult hypothalamus. Specifically, we examine precursor cell proliferation and survival in *Tph2^-/-^* mice at baseline and following Western-type diet (WD). Our results show a decline in BrdU numbers with aging in the absence of 5-HT. Furthermore, wild type mice under dietary challenge decrease cell proliferation and survival in the hypothalamic niche. In contrast, increased high-calorie food intake by *Tph2^-/-^* mice does not come along with alterations in cell numbers. However, lack of brain 5-HT results in a shift of cell phenotypes that was abolished under WD. We conclude that precursor cells in the hypothalamus retain fate plasticity and respond to environmental challenges. A novel link between 5-HT signaling and cell genesis in the hypothalamus could be exploited as therapeutic target in metabolic disease.

## Introduction

Besides the well-known neurogenic niches, the subventricular zone and the subgranular zone of the dentate gyrus, the hypothalamus has emerged as third region of postnatal neurogenesis and gliogenesis (Kokoeva et al., 2005; Lee et al., 2012; Robins et al., 2013a; Chaker et al., 2016). Positioned between the third ventricle and the median eminence, specialized radial glial tanycytes are thought to regulate the hypothalamic in- and output of hormones and nutrients to maintain body homeostasis (reviewed in Bolborea and Dale, 2013). Cell genesis within the adult hypothalamus may have an important role in feeding and reproduction control, in mediating stress responses, and in energy metabolism. Recent studies indicate the stem cell niche is responsive to mitogens (Haan et al., 2013; Robins et al., 2013a), leptin (Kokoeva et al., 2005) and dietary challenges (Lee et al., 2012, 2014), and thereby regulates appetite and energy expenditure (Pierce and Xu, 2010). The widespread monoamine serotonin (5-HT) impacts the variety of these functions (reviewed in Donovan and Tecott, 2013). Dysregulation of the 5-HT system leads to age-related memory loss and depression (reviewed in Alenina and Klempin, 2015), but also to changes in appetite and energy metabolism (Nigro et al., 2013; D’Agostino et al., 2018). At the same time, the precise role of 5-HT in the regulation of hypothalamic functions remains unknown. Here, we address this using tryptophan hydroxylase 2-deficient (*Tph2^-/-^*) mice selectively depleted of brain 5-HT. *Tph2^-/-^* mice exhibit transient early postnatal growth retardation, modest impairment of sleep and respiration, accompanied by a less anxious and highly aggressive phenotype (Alenina et al., 2009; Mosienko et al., 2012). Given the role of 5-HT in stress response and energy balance, we studied cell proliferation and fate plasticity in the hypothalamic nuclei of *Tph2^-/-^* mice, and the effect of high fat high cholesterol diet Western-type diet (WD) on cell survival. Our data reveal an age-dependent decline in BrdU numbers and alterations in food intake and cell phenotypes in the lack of brain 5-HT.

## Materials and Methods

### Animals and Treatment

*Tph2^-/-^* mice (Alenina et al., 2009) were bred onto the C57BL/6N background for more than 10 generations. Mice were housed under standard laboratory conditions with a light/dark cycle of 12 h each. Brain slices of young-adult (6 weeks of age, postnatal day (P) 42; *n* = 10), adult (3 months of age, P80; *n* = 8), and 1-year-old (P1y; *n* = 8) female mice and their littermates (CTR) (Klempin et al., 2013) were analyzed for baseline cell proliferation in the hypothalamus. Animals received three intraperitoneal injections (i.p.) of BrdU (5-bromo–2′-deoxyuridine, 50 mg/kg; Sigma-Aldrich) at 8:00 (2 h after lights went on), 14:00, and 20:00, and were killed 24 h after the first injection. Estrous cycle was not determined. Another cohort of three-month-old female *Tph2^-/-^* mice and their littermates (*n* = 20) were randomly assigned to two groups for standard diet (SD, 5.0% fat, 17.8% protein, 11.0% sugar) or WD (21.2% fat, 17.5% protein, 33.2% sugar, 2.071 mg/kg Cholesterol; TD-88137 ssniff, Germany). At day 6, 7, and 8 of the diet, BrdU (50 mg/kg) was injected twice per day. Animals were sacrificed after 7 weeks (49 days) of either diet to determine proliferation and survival of newly generated cells in the hypothalamus.

### Tissue Preparation and Immunohistochemistry

Mice were deeply anesthetized and perfused transcardially with 0.9% sodium chloride followed by 4% paraformaldehyde (PFA). Brains were removed and placed into PFA overnight, and transferred into 30% sucrose the following day. BrdU immunohistochemistry followed the peroxidase method in accordance with an established protocol (Klempin et al., 2013). Briefly, one in-six series of sequential 40 μm coronal sections were stained free floating, and all immunoreactive cells detected throughout the hypothalamus were counted. The mean of both hemispheres was taken, and the total number of BrdU-positive (BrdU+) cells was estimated by multiplying cell counts by six. For the first experiment, BrdU numbers were determined per hypothalamic nuclei (arcuate nucleus, median eminence, third ventricle, and ventromedial nucleus). For Ki67 and phenotypic analysis, one-in-twelve series of sections were labeled for multiple immunofluorescence staining. BrdU+ cells were evaluated using Keyence (BioRevo BZ-9000) or Leica TCS SP5 confocal microscope (Leica, Germany) for three-dimensional colocalization. Primary antibodies were applied in the following concentrations: anti-BrdU (rat, 1:500; Biozol/AbD serotec), anti-Glial Fibrillary Acidic Protein (GFAP; rabbit, 1:1000; Abcam), anti-Iba1 (rabbit, 1:500; Wako), anti-Ki67 (rat/mouse, 1:500; eBios), anti-NG2 (rabbit, 1:200; Abcam), anti-NeuN (rabbit, 1:500; Millipore), anti-RIP (rabbit, 1:1000; Merck), anti-S100β (mouse, 1:1000; SIGMA) and anti-Sox2 (goat, 1:1000; Santa Cruz Biotechnology); Alexa488-, Cy3-, or Cy5-conjugated secondary antibodies were used (1:250; Invitrogen, United States).

### Statistical Analysis

Statistical tests to detect differences between group means were performed by two-way ANOVA (experiment 1: genotype × age; experiment 2: genotype × diet), followed by Tukey’s *post hoc* tests, in cases where a significant *F* statistic was obtained (GraphPad PRISM 6.01 software). Student’s *t*-test was used for pair-wise comparison of BrdU and Ki67 analysis, and of hypothalamic subnuclei. All values are expressed as mean ± SEM. *P*-values of 0.05 were considered statistically significant.

## Results

### Cell Proliferation in the Adult Hypothalamus of *Tph2^-/-^* Mice Declines With Aging

**FIGURE 1:**
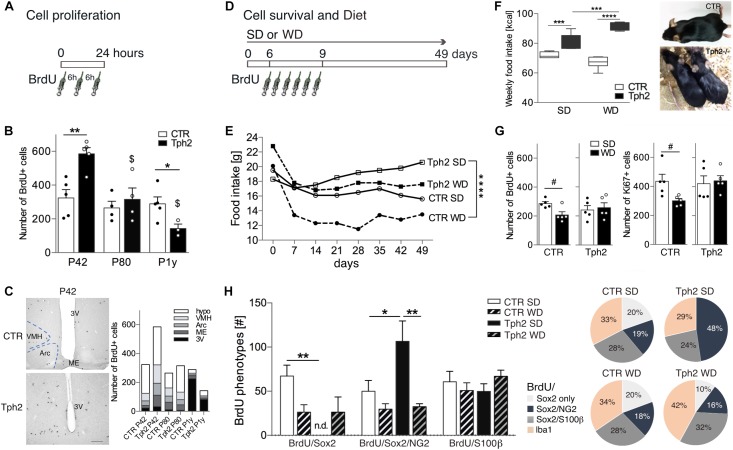
Cell proliferation, food intake and cell survival at baseline and after Western-type diet (WD). **(A)** The number of BrdU-positive (BrdU+) cells in the hypothalamic nuclei was quantified at 24 h after 3 injections of BrdU 6 h apart. **(B)** Cell proliferation (entire hypothalamus) was significantly higher in young-adult (P42) Tph2^-/-^ mice (Tph2), declined with aging and was significantly lower at 1 year of age (P1y) relative to littermates (CTR). **(C)** Representative images of BrdU peroxidase staining at P42, and cell numbers per hypothalamus (hypo; outside subnuclei) and per Arc, arcuate nucleus, VMH, ventromedial nucleus, ME, median eminence and 3V, third ventricle; Scale bar 150 μm. **(D)** Three-month-old Tph2 and littermates (CTR) were subjected to either standard diet (SD) or WD and BrdU was injected on days 6, 7, 8, twice per day, to determine cell proliferation and cell survival 6 weeks later. **(E,F)** Average daily food intake was least in CTR and largest in Tph2 (**E**, in g per cage of n = 5; **F**, in Kcal). Tph2 displayed oily and sticky fur upon WD. **(G,H)** At 49 days, WD resulted in a significant decrease in BrdU+ and Ki67+ cells in the hypothalamus of CTR, while it had no effect in Tph2 (**G**, Student’s *t*-test #p < 0.05). Decreased cell survival in CTR was reflected by a reduction in BrdU/Sox2+ cells, however, phenotype distribution was unaffected. In Tph2 under SD, half of cells co-express BrdU/Sox2/NG2, abolishing the number (graph) and percentage (pie diagram) of BrdU/Sox2-only cells **(H)**; notably, this was not observed after WD. Two-way ANOVA followed by Tukey’s *post hoc* test ^∗^p < 0.5, ^∗∗^p < 0.01, ^∗∗∗^p < 0.001, ^∗∗∗∗^p < 0.0001 between genotypes, ^$^p < 0.05 to P42 of the same genotype.

We first quantified baseline cell proliferation (Figure 1A) in the hypothalamus of *Tph2^-/-^* and CTR mice at different ages. In *Tph2^-/-^* mice, a dramatic decline in BrdU+ cells was observed with aging [*F*(2,20) = 9.786, *p _genotype__×_*_age_ = 0.0011; Figure 1B]. At P42, the number of BrdU+ cells was significantly higher relative to CTR (*p* = 0.0027; Figure 1B) while it was lower at P1y (*p* = 0.0431; Figure 1B). At P80, the amount of BrdU+ cells in the hypothalamus of CTR and *Tph2^-/-^* mice was similar (*p* = 0.5353; Figure 1B). When we looked at the distribution of BrdU+ cells within the various hypothalamic areas and nuclei (Figure 1C), the increase at P42 in *Tph2^-/-^* mice was reflected by increased cell numbers in the median eminence (Student’s *t*-test *p* = 0.0339) and arcuate nucleus (*p* = 0.0166), a tendency was observed for the ventromedial nucleus (*p* = 0.0668), while at P1y, *Tph2^-/-^* mice revealed significant fewer cells lining the third ventricle relative to CTR (*p* = 0.0057).

### Cell Survival Is Not Affected by Increased High-Calorie Food Intake in *Tph2^-/-^* Mice

Given the role in energy metabolism, we elucidated the effect of WD for 7 weeks on the number of newly generated cells in the hypothalamus of three-month-old *Tph2^-/-^* mice and littermates (Figure 1D). During the first week of WD, food consumption [g] declined but then remained constant for each group over time (Figure 1E). Average food intake [g] was least in CTR fed WD and largest in *Tph2^-/-^* mice fed SD [*F*(3,21) = 21.06, *p* < 0.0001; Figure 1E]. Translated to calorie intake (Kcal), *Tph2^-/-^* mice consumed more of both diets, most of the WD [*F*(1,23) = 27.99, *p _genotype × diet_* < 0.0001; Figure 1F]. *Tph2^-/-^* mice displayed oily and sticky fur starting at day 3 of WD that was absent in CTR (Figure 1F). Seven weeks of WD resulted in significantly decreased survival of BrdU+ cells in the hypothalamus of CTR (SD vs. WD, Student’s *t*-test *p* = 0.0154; Figure 1G). In contrast, increased high-calorie consumption by *Tph2^-/-^* mice did not affect cell numbers (SD vs. WD, Student’s *t*-test *p* = 0.7351; Figure 1G). Cell proliferation at 49 days was assessed by Ki67-labeling; here too, WD significantly decreased the number of newly generated cells in CTR (Student’s *t*-test *p* = 0.0284) while no effect was observed in *Tph2^-/-^* mice (Student’s *t*-test *p* = 0.7665; Figure 1G).

### Lack of Brain 5-HT Affects Phenotype Distribution

**FIGURE 2:**
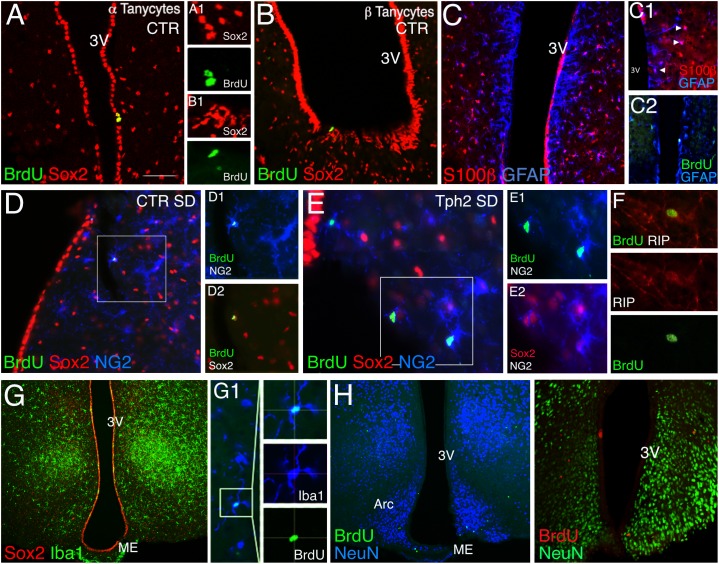
Immunohistochemistry of cell phenotypes. **(A,B)** In CTR mice, a sparse number of BrdU/Sox2-tanycytes was found lining the third ventricle and was absent in Tph2^-/-^ mice (Tph2; α tanycyte, **A1**, β tanycyte, **B1**) Scale bar 150 μm. **(C)** GFAP+ cells lining the dorsal part of the ventricle often co-express S100β, arrowheads **(C1)**, and were rarely dividing (BrdU/GFAP+, **C2**). **(D–F)** BrdU/Sox2/NG2+ cells account to ∼20% in CTR **(D)** and ∼50% in Tph2 **(E)** at standard diet, SD. Approx. 30% of these cells followed the oligodendrocyte fate expressing the lineage marker RIP **(F)**. **(G)** One third of BrdU+ cells adopted a microglia fate in all groups (Iba1+/Sox2-). **(H)** No BrdU/NeuN co-expressing cell was found in all groups at 49 days of the experiment. Arc, arcuate nucleus, ME, median eminence, and 3V, third ventricle.

We next assessed phenotypes of BrdU+ cells (Figure 1H) to determine the cell type affected in the absence of 5-HT and upon WD. BrdU+ cells were co-stained for the neural stem/progenitor markers GFAP and Sox2 (SRY-related HMG-box gene 2), the proteoglycan NG2, the oligodendrocyte lineage marker RIP, the astrocytes marker S100β, and the microglia marker Iba1. Sox2+ cells were highly proliferative; lining the third ventricle, they were considered as tanycytes (Figure 2A,B). Our results show a remarkably sparse number of BrdU-expressing tanycytes in CTR independent of the diet (<2.0% of total BrdU). Notably, no BrdU/Sox2-tanycyte was observed in *Tph2^-/-^* mice. GFAP+ cells located along the dorsal part of the ventricle (α2 tanycytes, (Robins et al., 2013a); Figure 2C) often co-expressed S100β (Figure 2C1), and were rarely dividing (Figure 2C2). BrdU/GFAP+ cells were only seen at SD for both genotypes accounting to 4% in CTR, and 5.8% of total BrdU in *Tph2^-/-^* mice (Figure 2C2). Analysis of glial phenotype distribution revealed two-thirds of BrdU+ cells expressed Sox2 (Figure 1H). In CTR, cells were predominantly triple labeled for BrdU/Sox2/S100β (∼30%) and BrdU/Sox2/NG2 (∼20%; Figure 1H, 2D). Notably, all BrdU/S100β+ cells co-expressed Sox2. In *Tph2^-/-^* mice at SD conditions, half of the cells co-expressed BrdU/Sox2/NG2 [*F*(2,46) = 7.764, p_ genotype_ = 0.0012; Figure 1H, 2E], lowering the amount of BrdU/Sox2-only cells. After WD, the decrease in BrdU+ cell numbers in CTR was reflected by decreased double and triple labeling of glial markers with a significant reduction in BrdU/Sox2+ cells (SD vs. WD, *p* = 0.024; Figure 1H). However, phenotype distribution was unaffected by WD in CTR. In *Tph2^-/-^* mice upon dietary challenge, the large population of BrdU/Sox2/NG2+ precursor cells was diminished to 16%, and fate distribution among glial cells was similar to CTR (Figure 1H, pie diagram). Approximately 30% of cells in all groups adopted an oligodendrocyte fate co-expressing BrdU/RIP (Figure 2F). Another third of BrdU+ cells (Sox2-) adopted a microglia fate characterized by co-expression of Iba1 (Figure 2G). We did not observe BrdU/NeuN+ cells in any group (Figure 2H). Congruent to NeuN staining, no BrdU+ cell expressing the neuronal marker β-III-Tubulin was found (Tuj1; not shown).

## Discussion

Our data demonstrate an age-dependent decline in cell proliferation in the hypothalamus of *Tph2^-/-^* mice, but not in CTR, and a large population of proliferating Sox2/NG2 cells. In the lack of brain 5-HT, no proliferating Sox2-tanycytes were detected and the high-calorie-induced decrease in cell proliferation and survival was absent. Furthermore, we did not find newly generated neurons at 6 weeks after BrdU but a diet-induced altered phenotype distribution in *Tph2^-/-^* mice.

Neural stem/progenitor cell populations of α and β tanycytes in the adult hypothalamus, and postnatal born neurons, contribute to brain plasticity and potentially respond to hormones and nutrition to maintain metabolic rate (Lee et al., 2012; Bolborea and Dale, 2013; Haan et al., 2013; Robins et al., 2013a). Serotonin is involved in the regulation of feeding and metabolism, e.g., food intake enhances hypothalamic 5-HT indicating satiety (Leibowitz and Alexander, 1998; Schwartz et al., 2015; Nectow et al., 2017). On cellular level, 5-HT2C receptors on POMC neurons might particularly mediate the 5-HT effects (Lam et al., 2008; D’Agostino et al., 2018). Our data show that in the absence of brain 5-HT, *Tph2^-/-^* mice consumed significantly more suggesting they might lack satiety. This might correspond to no detectable proliferating Sox2-tanycytes; in a next step, POMC neurons in *Tph2^-/-^* mice should be studied (Klein et al., 2019). Furthermore, in the lack of brain 5-HT, a different subpopulation of precursor cells, Sox2/NG2, is favored and affected by WD; indicating 5-HT plays an important role at the stem/progenitor cell stage. Our earlier findings on neurogenesis in the hippocampus of *Tph2^-/-^* mice also revealed changes at Sox2 cell stage, suggesting physiological adaptations to changes in 5-HT supply to maintain homeostasis (Klempin et al., 2013). Hypothalamic Sox2/NG2 or NG2-only cells are highly proliferative with glial and some neuronal potential (Robins et al., 2013b). Fate plasticity makes them favorably affected to changes in the environment and in turn might act as compensatory mechanism. Increased proliferation of Sox2/NG2+ cells in *Tph2^-/-^* mice might compensate for the lack of BrdU/Sox2-tanycytes. Although a small number of newborn neurons was reported in few lineage tracing studies (Lee et al., 2012; Robins et al., 2013a) we have not detected any using our treatment protocol–based on BrdU and available markers of lineage progression–and it remains speculative whether the high number of NG2 cells eventually become neurons (Robins et al., 2013b).

In CTR mice, BrdU/Sox2+ cells were affected upon high-calorie consumption, reducing this precursor subset. In aged CTR mice, no difference in cell proliferation was observed; and a decline in only Sox2-tanycytes, e.g., starting at 11 months of age, was reported earlier (Zhang et al., 2017). In *Tph2^-/-^* mice, lack of brain 5-HT *per se* might not be responsible for the age-dependent decline in cell proliferation but overall changes in tanycyte and precursor numbers which in turn lead to reduced BrdU detected at P1y.

In this study, we explored the novel neural stem cell niche of the adult hypothalamus and analyzed the role of brain 5-HT in food intake and cell genesis. We show that precursor cells in the hypothalamus retain fate plasticity and respond to both intrinsic changes and environmental stimuli.

## Ethics Statement

All procedures were approved by LAGeSo (Berlin, Germany) and carried out in accordance with the European Communities Council Directive 2010-63 UE.

## Author Contributions

MvL and FK designed the research, analyzed data and wrote the manuscript. MvL, MS, and FK performed experiments. NA bred and provided *Tph2^-/-^* animals.

## Conflict of Interest Statement

The authors declare that the research was conducted in the absence of any commercial or financial relationships that could be construed as a potential conflict of interest.
